# Branching pattern and morphogenesis of medusa tentacles in the jellyfish *Cladonema pacificum* (Hydrozoa, Cnidaria)

**DOI:** 10.1186/s40851-019-0124-4

**Published:** 2019-03-14

**Authors:** Akiyo Fujiki, Shiting Hou, Ayaki Nakamoto, Gaku Kumano

**Affiliations:** Asamushi Research Center for Marine Biology, Graduate School of Life Sciences, Tohoku University, 9 Sakamoto, Asamushi, Aomori, 039-3501 Japan

**Keywords:** Adhesive organ, Branching morphogenesis, *Cladonema pacificum*, Cnidarian, Jellyfish, Medusa tentacle, Mesoderm, Nematocyst, RTK signaling

## Abstract

**Background:**

Branched structures are found in many natural settings, and the molecular and cellular mechanisms underlying their formation in animal development have extensively studied in recent years. Despite their importance and the accumulated knowledge from studies on several organs of *Drosophila* and mammals, much remains unknown about branching mechanisms in other animal species. We chose to study the jellyfish species *Cladonema pacificum*. Unlike many other jellyfish, this species has branched medusa tentacles, and its basal phylogenetic position in animal evolution makes it an ideal organism for studying and understanding branching morphogenesis more broadly. Branched tentacles are unique compared to other well-studied branched structures in that they have two functionally distinct identities: one with adhesive organs for attaching to a substratum, and another with nematocyst clusters for capturing prey.

**Results:**

We began our analyses on *C. pacificum* tentacles by observing their branching during growth. We found that tentacle branches form through repeated addition of new branches to the proximal region of the main tentacle while it is elongating. At the site of branch bud formation, we observed apical thickening of the epidermal epithelial layer, possibly caused by extension of the epithelial cells along the apico-basal axis. Interestingly, tentacle branch formation required receptor tyrosine kinase signaling, which is an essential factor for branching morphogenesis in *Drosophila* and mammals. We also found that new branches form adhesive organs first, and then are transformed into branches with nematocyst clusters as they develop.

**Conclusions:**

These results highlight unique features in branch generation in *C. pacificum* medusa tentacles and illuminate conserved and fundamental mechanisms by which branched structures are created across a variety of animal species.

## Background

During organ development in animals and plants, branched structures form to expand epithelial surface areas and maximize functions. Such branched structures include *Drosophila* trachea [1, 2], plant leaf veins [3], and mammalian lungs [4, 5], kidneys [6, 7], pancreas [8, 9], salivary glands [10, 11], mammary glands [12, 13], and blood vessels [14]. Marine colonial organisms, such as corals, bryozoans, and hydroids, are also branched structures [15–18]. Although these structures appear to be morphologically diversified, recent molecular and cellular studies of branching morphogenesis, mainly in *Drosophila* and mammals, have highlighted the common and fundamental principles of organ branch formation. For example, complex and elaborated branched organs are created by the repeated application of a simple branching rule occurring at the tip of the branching structures (e.g. [7, 19, 20]). In addition, receptor tyrosine kinase (RTK) signaling, such as fibroblast growth factor (FGF) signaling, is known to stimulate cellular morphogenesis processes, such as cell migration and proliferation, which are required for branch formation in most of the branched organs [20–22]. However, how widely these mechanisms are conserved across animal species remains undetermined. Furthermore, it is unclear whether branched organs can be created by different mechanisms, as the current knowledge of molecular and cellular mechanisms of branching morphogenesis relies mostly on studies from *Drosophila* and mammals [21–23].

**Fig. 1:**
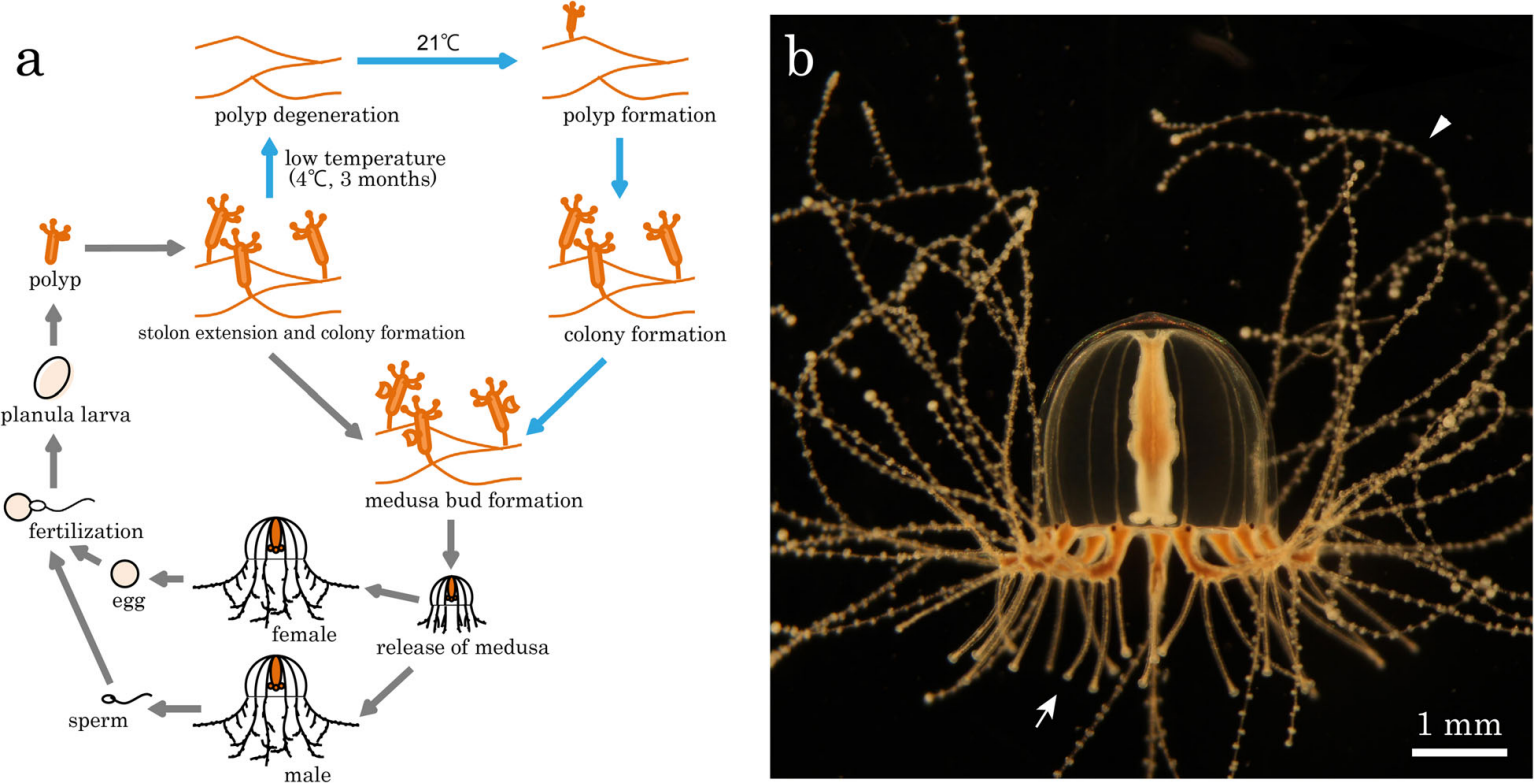
The *Cladonema pacificum* jellyfish. **a** Life cycle of *C. pacificum*. An individual polyp generated through metamorphosis of the planula larva extends a stolon and produces genetically identical populations of polyps along the extended connection. Polyps are full of nutrients and form medusa buds on the side of their bodies, and they are eventually released and grow into mature female and male organisms. Eggs and sperm spawned by light stimuli are fertilized, and the fertilized eggs develop into planula larvae. The cycle shown by blue arrowheads indicates an artificial method commonly used in the laboratory to increase the efficiency of medusa formation. In this method, polyps are kept at 4 °C for at least three months resulting in polyp degeneration, and then they again transferred back at 21 °C to induce the formation of new polyps. **b** An adult *C. pacificum*. The medusa tentacles of this species are branched into two different types with either nematocyst clusters along their length for hunting (arrowhead) or adhesive organs on their tips for landing (arrow). Scale bar: 1 mm

The jellyfish *Cladonema pacificum* is a hydrozoan species belonging to the phylum Cnidaria. This species is found along coastal areas of Japan. The medusa has an umbrella of approximately 3 mm in diameter, is benthic, and inhabits seawater while adhering to seagrass most of the time. This species has been cultivated from generation to generation in the laboratory [24, 25] (Fig. 1a) and thus is an ideal cnidarian species to study different aspects of biology, such as egg maturation upon light stimulation [26]. One of the key characteristics of *Cladonema* is that its medusa tentacles are branched (Fig. 1b). Branched tentacles are rare among Medusozoa, although they are commonly found in the jellyfish of the Cladonematidae family [27], and are considered to be evolutionarily derived characters. These branches differentiate into two functionally distinct types: one specified for prey-capturing, which bears nematocyst clusters along the length of the branches, and another specified for attaching to a substratum, such as seagrass (landing), through adhesive organs at the tip of each branch. Branching in *Cladonema* medusa tentacles may thus be a complex process involving identity differentiation in addition to branch formation.

In the present study, we continuously monitored the same medusa tentacles of *C. pacificum* for one month. Every 24 h, we observed and recorded their branching patterns during their growth phase and included measurements on how they branched and how the two branch types were differentiated. We also observed branch bud-forming epithelial cells using confocal microscopy. Finally, we analyzed the mechanisms of branch formation with an inhibitor treatment and of branch differentiation with branch ablation experiments. Our results provide fundamental descriptive information on tentacle branch patterning in *C. pacificum*, and indicate that it has both conserved and unique mechanisms compared to other branching systems, such as those in *Drosophila* and mammals.

## Materials and methods

### Animals

The UN2 line of *Cladonema pacificum* jellyfish [26] was used in this study. It was originally harvested near the island of Urato Nono-shima in Miyagi Prefecture, Japan. UN2 animals are male and spawn sperm upon dark stimuli [25]. They are kept in small containers filled with filtered seawater (FSW) at 21 °C in the laboratory and fed everyday with an excess amount of *Artemia salina* Nauplius (A & A Marine Brine Shrimp Eggs, Vietnam) unless otherwise specified. The FSW is replaced after feeding to keep it clean. The life cycle of this jellyfish species is shown in Fig. 1a.

### Observation of tentacle formation

**Fig. 2:**
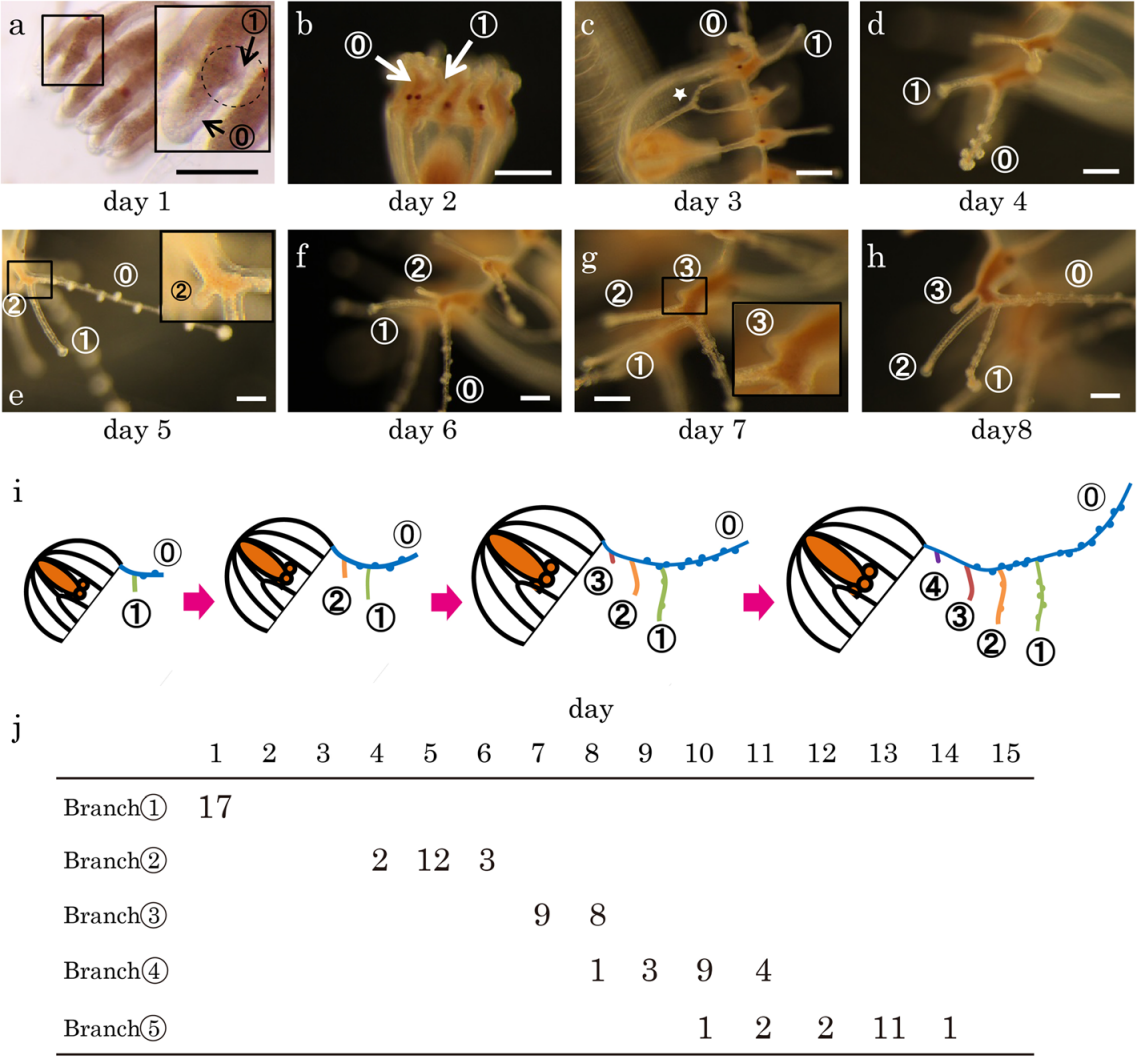
The process of tentacle branch formation. **a**–**h** Images of the same tentacle with branches forming as it grows. The main tentacle that extends from the medusa umbrella is numbered as ⓪. Newly formed branches are numbered in the order of their formation (the first branch = Branch①, the second branch = Branch②, the third branch = Branch③ …). These numbers are circled in this figure and others. The day when the first branch is observed (① in A) is defined as Day 1. In most cases, the medusa is released on Day 3. An example of a branched radial canal used as a reference to continually track same tentacle is marked by a star (**c**). The insets in **a**, **e** and **g** are blown-up images of the rectangle regions shown in the respective images. Scale bars: 200 μm. **i** Summary of tentacle branch formation. New branches are formed one after another at positions proximal to the older branches on the main tentacle (blue) as it elongates and are shown in different colors. **j** A table showing when branches were formed in 17 cases/tentacles. Each number represents how many numbered branches were observed for the first time on a given day among 17 tentacles

The same tentacles of the same medusa animals were tracked for 1 month, and their branch patterns and whether they formed adhesive organs and/or nematocyst clusters were observed and recorded every 24 h using a stereomicroscope (SZX16, Olympus). The medusae with branched radial canals (Fig. 2c) were selected for observation, and the branched canals were used as references to keep track of the same tentacles. These medusae were reared individually. Only those tentacle branches confirmed to have functional adhesive organs or nematocysts were counted as having the respective tissue type. The presence of functional adhesive organs was determined by examining the branch’s ability to stick to a tungsten needle, and the presence of nematocysts was evaluated by examining the branch’s ability to capture *Artemia* Nauplius. The day when the first branches were formed was defined as Day 1. On Day 1, the medusae were still attached to the side of the polyps (Fig. 1a).

### Phallacidin staining

Day 5 medusae were relaxed before fixation by gradually adding drops of 0.4 M MgCl_2_ solution into the sea water until they were immobilized. The relaxed medusae were then subjected to perfusion fixation by gradually dropping 0.2% formalin-containing phallacidin buffer (100 mM PIPES, pH 6.9, containing 400 mM sucrose and 50 mM EGTA) into the sea water. Although they began moving soon after the perfusion fixation began, they were eventually immobilized again. Once immobilized, the medusae were incubated for 15 min and fixed in 4% formalin in phallacidin buffer for an additional hour. The fixed medusae were then washed three times with phosphate-buffered saline (PBS). To facilitate staining and observation, the proximal portions of the main tentacles containing the forming buds of the second branches (referred to in Figures as Branch②) were mainly used, and other parts, including the umbrella, the distal parts of the main tentacles, and the first branches (referred to in Figures as Branch①), were excised to the extent possible using a razor.

A staining solution with phallacidin was prepared at 5 units/ml by dissolving air-dried BODIPY FL Phallacidin (Molecular Probes) in PBS and 0.2% TritonX-100. The dissected proximal portions of the tentacles were incubated in the staining solution for one hour at room temperature and then washed three times for 10 min each in PBS. The stained specimens were mounted in VECTASHIELD (Vector Laboratories) and observed through a confocal microscope (LSM5 Pascal, Zeiss). Images were Z-stacked with ImageJ. The abaxial-adaxial axis (the outer-inner surfaces) of the main tentacle was recognized using the eye spot as a reference for the abaxial side.

**Fig. 3:**
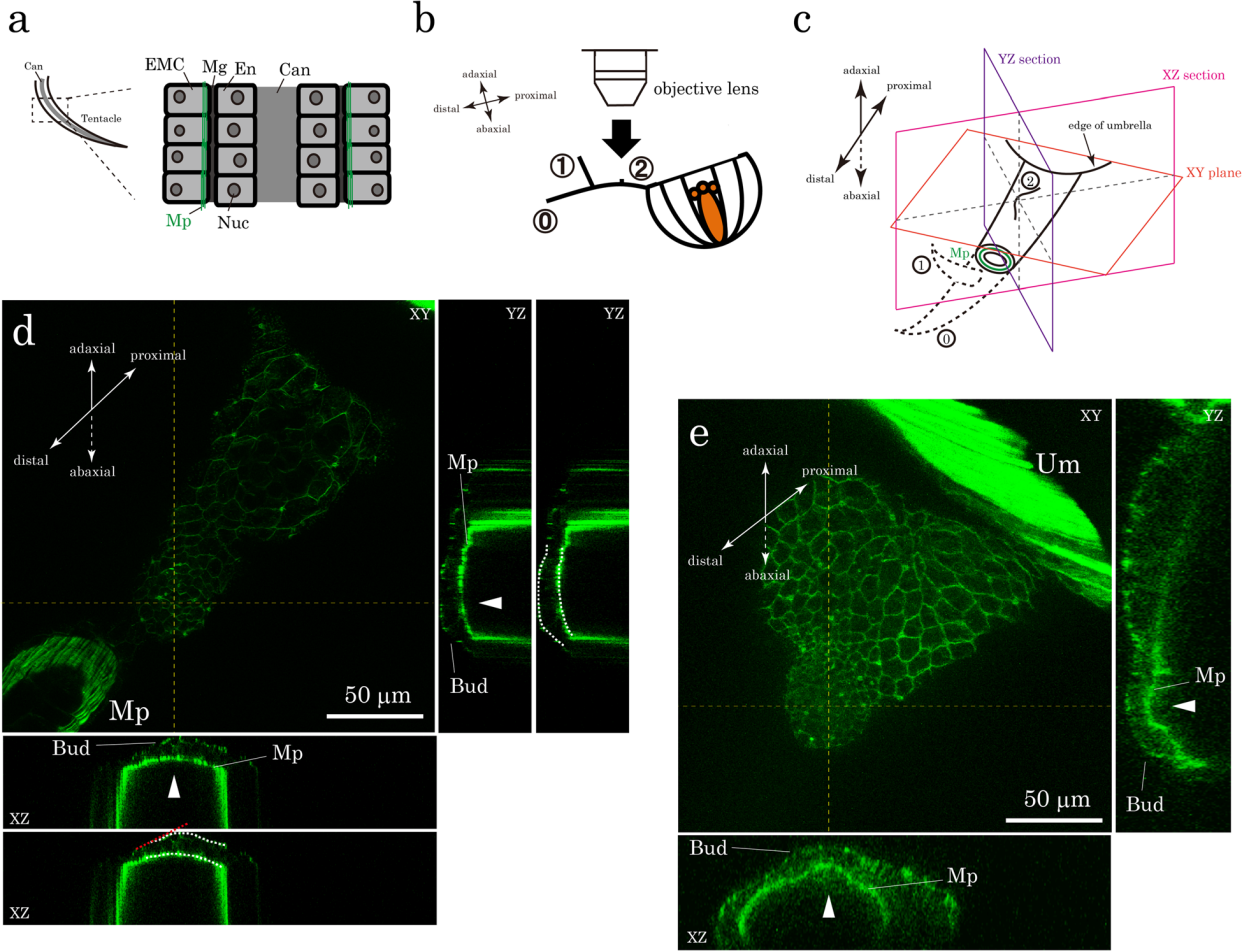
Morphology of branch buds. **a** A diagram of sagittal section of the main tentacle is shown. The tentacle is a tube-like structure mainly composed of two layers of epithelial cells: outer epidermal epithelio-muscular cells (EMCs), which have basal myoid processes containing contractile muscle fibrils running longitudinally shown in green (Mp), and inner endoderm cells (En) [28]. These two cell layers are separated by a jelly-like substance called mesoglea (Mg). Other components, such as nematocytes and interstitial cells, are omitted for simplicity. Can: canal, Nuc: nucleus. **b, c** Diagrams showing how the branch buds were viewed in **d** and **e**. The tentacles are oriented with their adaxial sides up. The distal portion of the tentacle, which was cut off when observed, is outlined in broken line in **c**. ⓪: the main tentacle; ①: the first branch; ②: the branch bud forming the second branch. Mp: myoid processes in the section of the tentacle. **d**, **e** Confocal images for the buds of the second branch (Branch②) stained with Phallacidin. Adaxial views. These images are generated from multiple Z-sections processed with maximum intensity projections. The tips of the buds were roughly estimated by the highest points of the EMC layers shown in the XZ (under the image) and YZ (right side of the image) sections and are indicated by the points of intersection between the vertical and horizontal yellow broken lines. The yellow lines also indicate the positions of the XZ and YZ sections. The same two images for the XZ and YZ sections in **d** are shown with ones outlining the apical and basal sides of the EMC layers (white broken lines). Buds in **d** and **e** are at different stages of bud formation. Mp: myoid processes. Um: medusa umbrella. Scale bars: 50 μm.

**Fig. 4:**
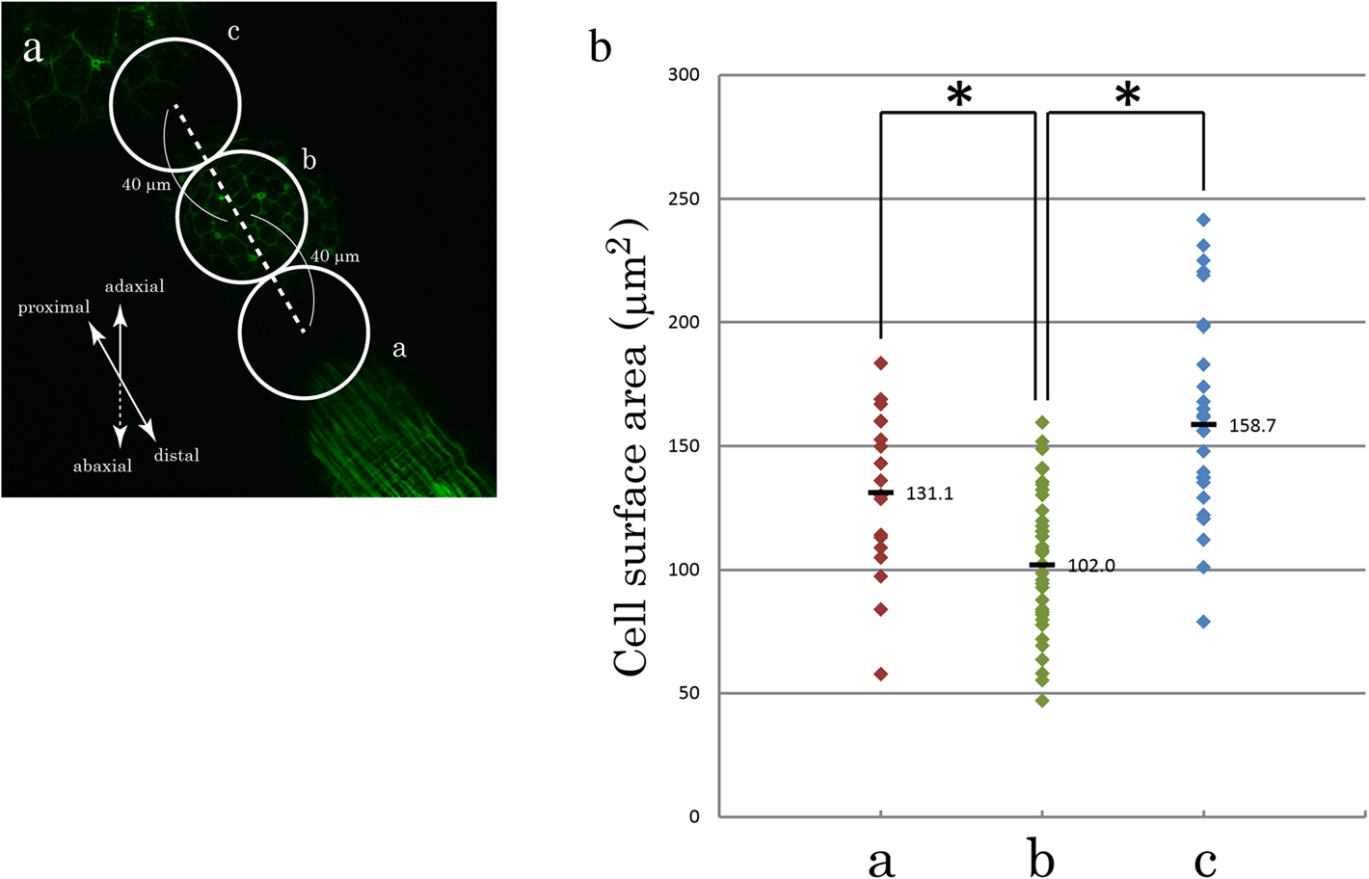
**a** An image showing the fields in which the apical surface areas of EMCs were calculated. The tentacle is viewed adaxially according to Fig. 3b. The position of area b was determined as a circle centered at the estimated tip of the branch bud with a radius of 20 μm. The areas a and c, with the same shape and size as the area b, were in distal and proximal locations, respectively, and were in contact with area b along the length of the tentacle. The cell surface areas of all strongly phallacidin-stained cells in these three areas were calculated from the images of the XY planes using ImageJ. Only the image focused on area b is shown in this figure. Different Z sections of the same samples were used for calculation of the areas a and c. **b** The measured values are shown. For the cells in areas a and c in **a**, the values obtained from the XY planes are used (“a” and “c” on the horizontal axis); in contrast, for those in area b in **a**, the maximum extent possible of the surface area values was estimated from the calculated values from the XY planes, taking the angle created by the bud bulging into account (see Materials and methods), and is used (“b” on the horizontal axis). The results from five different branch buds at the stage that corresponds to Fig. 3d are shown. * *P* < 0.005 (Welch t-test). The average values are 131.1 μm^2^ (area a), 102.0 μm^2^ (area b), 158.7 μm^2^ (area c) and are indicated by black bars

The maximum extent possible of the apical surface area values of the epidermal epithelial cells in the branch buds (area b in Fig. 4a) was estimated from the calculated values from the XY planes (see Fig. 4 legend). The maximum angle of the apical side of the epidermal epithelial layer in the bud with respect to the XY plane was measured using the cross sections of the bud (e.g. red broken line in the second XZ section in Fig. 3d), and was found to be 31.9° in average (*n* = 4). Accordingly, the surface area values obtained from the XY planes for area b were multiplied by {1/cos (31.9π/180)}^2^ for the maximum estimation, in which all the cells in area b constituting the buds are estimated to have an angle of 31.9° and be regarded as taking round shape.

### Inhibitor treatment

Medusae were treated with 10 μM MEK inhibitor UO126 (Calbiochem) either from Days 4 to 6 or from Days 6 to 8. Medusae form second (Branch②) and third branches (referred to in Figures as Branch③) by Days 6 and 8, respectively (Fig. 2j). To minimize the effect of the inhibitor on tentacle growth itself, feeding was ceased during the inhibitor treatment. The formation of second or third branches was evaluated on Day 6 or 8, respectively.

### DiI labeling

CellTracker CM-DiI (Molecular Probes) was dissolved at 2 mg/ml in soy oil and was centrifuged to remove debris before it was used for labeling. The cuticle-like structure covering the tentacle branches prevented use of the conventional labeling technique of placing a drop of DiI solution on the surface of the targeted object. Therefore, the tip of a glass capillary needle containing the DiI solution was pricked into the branches while a drop of the DiI solution was injected. The third branches (Branch③) (Fig. 2) were labelled on Day 7 and tracked by continuously observing them through a fluorescence microscope (BX53, Olympus) until Day 14.

### Branch ablation

Medusae were relaxed with gradual addition of drops of 0.4 M MgCl_2_ solution before ablation. The distal parts of the medusa tentacles including the first branches (Branch①) were removed on Day 5 or 6 by cutting the main tentacles at the position between the first and second (Branch②) branches with a razor (Fig. 8a), leaving the second branches attached to the main body. The second branches had not yet acquired functional adhesive organs when the ablation was carried out either on day 5 or 6. After ablation, the medusae were put back in the normal FSW and woken, and the remaining second branches were observed every 24 h to examine whether they had acquired adhesive organs and/or nematocysts.

## Results

### Branching pattern and morphogenesis of medusa tentacles

To understand how the tentacles of *Cladonema pacificum* medusae branch, we monitored the growth of tentacles every 24 h after the first branches (Branch①) were formed and recorded branching patterns for 15 days (Fig. 2a-h, j). We found that new branches are formed one after another on the main tentacle (referred to in Figures as ⓪) at positions proximal to the branches previously formed (Fig. 2i), such that the youngest branches are always located most proximally. During branch formation, the main tentacles continuously extended in length and pushed the newly formed branches away from their proximal ends (Fig. 2i). The branches were always formed on the adaxial side of the main tentacle (Fig. 2a-h). Five branches (Branch① to ⑤ in the order of their formation) were formed during the 15-day period. Once each branch was formed, it did not form additional branches. These results indicate that *C. pacificum* medusa tentacles branch through repeated addition of new branches in the proximal region of the main tentacles.

We next observed cell morphological changes during the initial phase of branch formation. We looked at small branch buds growing into second branches (Branch②) on Day 5 with confocal microscopy after staining the cells with phallacidin (Fig. 3b). Since second branches were first observed from Day 4 to 6, with most forming on Day 5 (Fig. 2j), we expected to observe different stages of bud formation on Day 5. Contractile muscle fibrils exist at the basal end of the tentacle epidermal epithelial cells in the epithelio-muscular cells (EMCs) (Fig. 3a) [28]. Muscle fibrils stain strongly with phallacidin, and we were able to visualize the shape of the epidermal epithelial cell layer around the forming buds in cross sections of the YZ and XZ planes of confocal images (Fig. 3c, d, e). In some cases (*n* = 5), we observed that both the apical and basal sides of the epidermal outer layer bulged outward in the buds (arrowheads in Fig. 3e). In others (n = 5), only the apical sides bulged, while the basal sides remained moderately curved along the shape of the tentacles (arrowheads and white broken lines in Fig. 3d). Considering that these buds eventually grow into branches with a tube-like structure similar to that of the main tentacles (Fig. 3a), the buds with only the apical bulging were probably fixed at an earlier stage of branch formation.

To gain further insight into the cell morphology during branch bud formation, we measured the apical surface areas of the EMCs constituting the forming buds (area b in Fig. 4a) and of those away from the buds (areas a and c in Fig. 4a). In this analysis, we realized that the direct measurement of the surface areas of the cells in the buds (area b) from the confocal images of the XY plane undervalues actual numbers due to the surface angle with respect to the XY plane created by the bud bulging. Therefore, we decided to estimate the maximum possible extent of the area values for the cells in the buds (see Materials and methods) and compare these values from values outside of the buds. We found that the maximally estimated values were still significantly smaller than values from the cells away from the buds (Fig. 4b), indicating that the apical surface areas of the cells in the buds are smaller than those away from the buds at the stage shown in Fig. 3d. The confocal observations shown here suggest that the initial step of branch formation may be apical thickening of the epidermal epithelial cell layer by the extension of the EMCs along the apico-basal axis.

### MEK signaling in branch formation

**Fig. 5:**
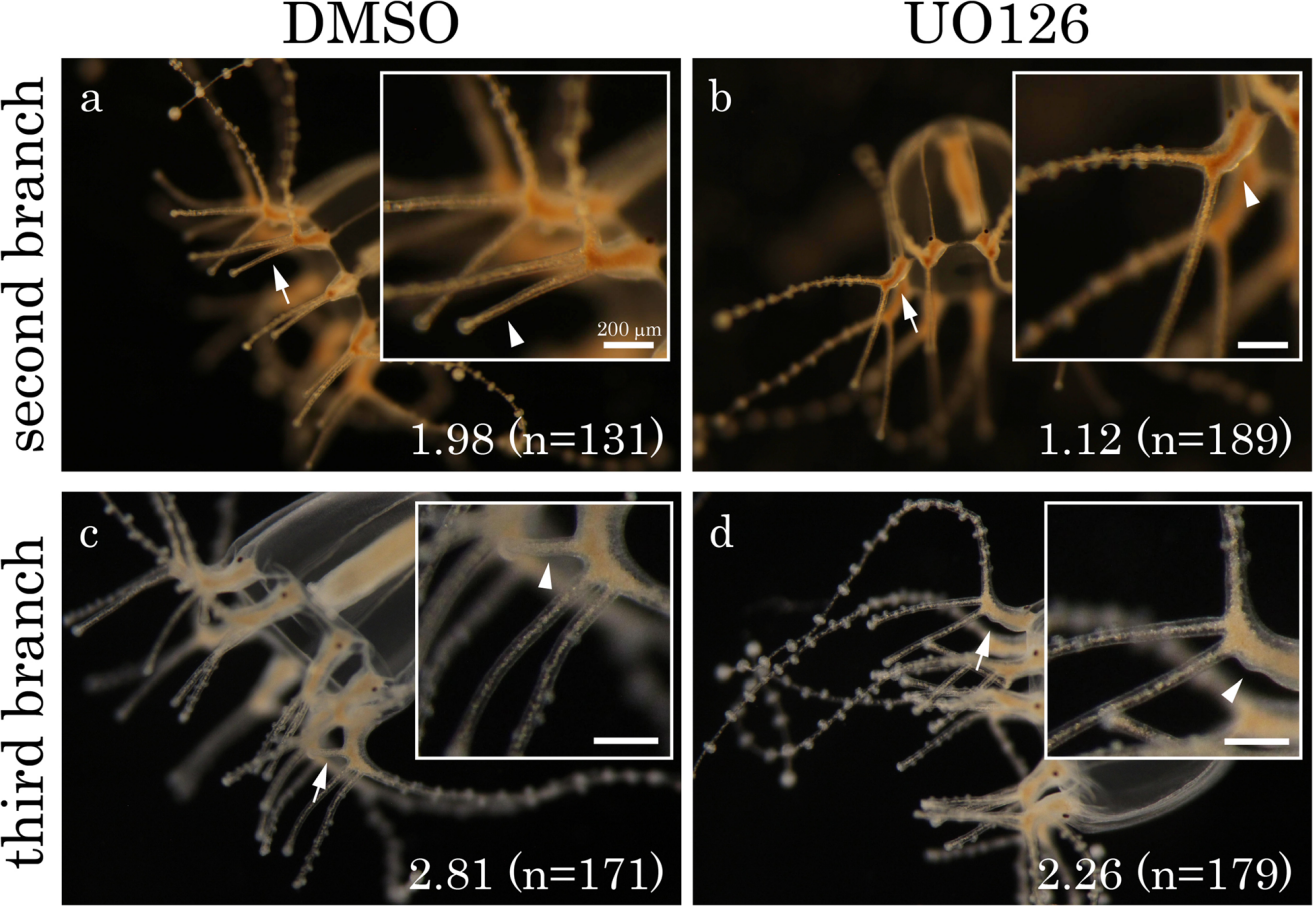
Effect of MEK inhibitor on branch formation. **a**–**d** Tentacle images of a control (**a**, **c**) and a UO126-treated (**b**, **d**) medusa on Days 6 (**a**, **b**) and 8 (**c**, **d**). The insets in the images are blown-up from the regions indicated by arrows. Arrowheads represent the presence (**a**, **c**) or absence (**b**, **d**) of the second (**a**, **b**) (Branch②) and third (**c**, **d**) (Branch③) branches. The mean numbers of branches observed are indicated in the right-bottom corner of the images. Samples were treated with DMSO or UO126 for two days either from Day 4 to 6 (**a**, **b**) or from Day 6 to 8 (**c**, **d**), during which most of the second or third branches, respectively, form normally (Fig. 2j). Feeding was terminated before drug treatment to prevent tentacle growth from affecting the results. Scale bars: 200 μm

To understand the molecular mechanisms by which medusa tentacles become branched, we treated growing tentacles with the MEK inhibitor UO126 for two days starting either on Day 4 or 6 and examined the effect on formation of second or third branches, respectively. MEK is a cytoplasmic component that transduces receptor tyrosine kinase (RTK) signaling in the signal-receiving cells [29]. Our earlier results showed that all the second and third branches were formed by Days 6 and 8, respectively (Fig. 2j). In this experiment, we did not feed the medusae during the inhibitor treatment and tried to minimize the effect of the inhibitor on tentacle growth. We found that the average number of branches, excluding the main tentacle, on Day 6 or 8 after inhibitor treatment was 1.12 (*n* = 189) or 2.26 (*n* = 179), respectively, indicating that most of the tentacles did not form second or third branches (arrowheads in Fig. 5b, d). In contrast, most of the control DMSO-treated tentacles had two (the average number was 1.98 (*n* = 131)) on Day 6 or three (the average number was 2.81 (*n* = 171)) branches on Day 8, and the second and third branches formed normally (arrowheads in Fig. 5a, c). Overall morphologies of UO126- and DMSO-treated tentacles were similar on Days 6 and 8 (Fig. 5). Although we cannot exclude the possibility that the MEK inhibitor affected other events and indirectly blocked branch formation, these results suggest that MEK and RTK signaling are involved in branch formation in medusa tentacles.

### Differentiation of the tentacle branches

**Fig. 6:**
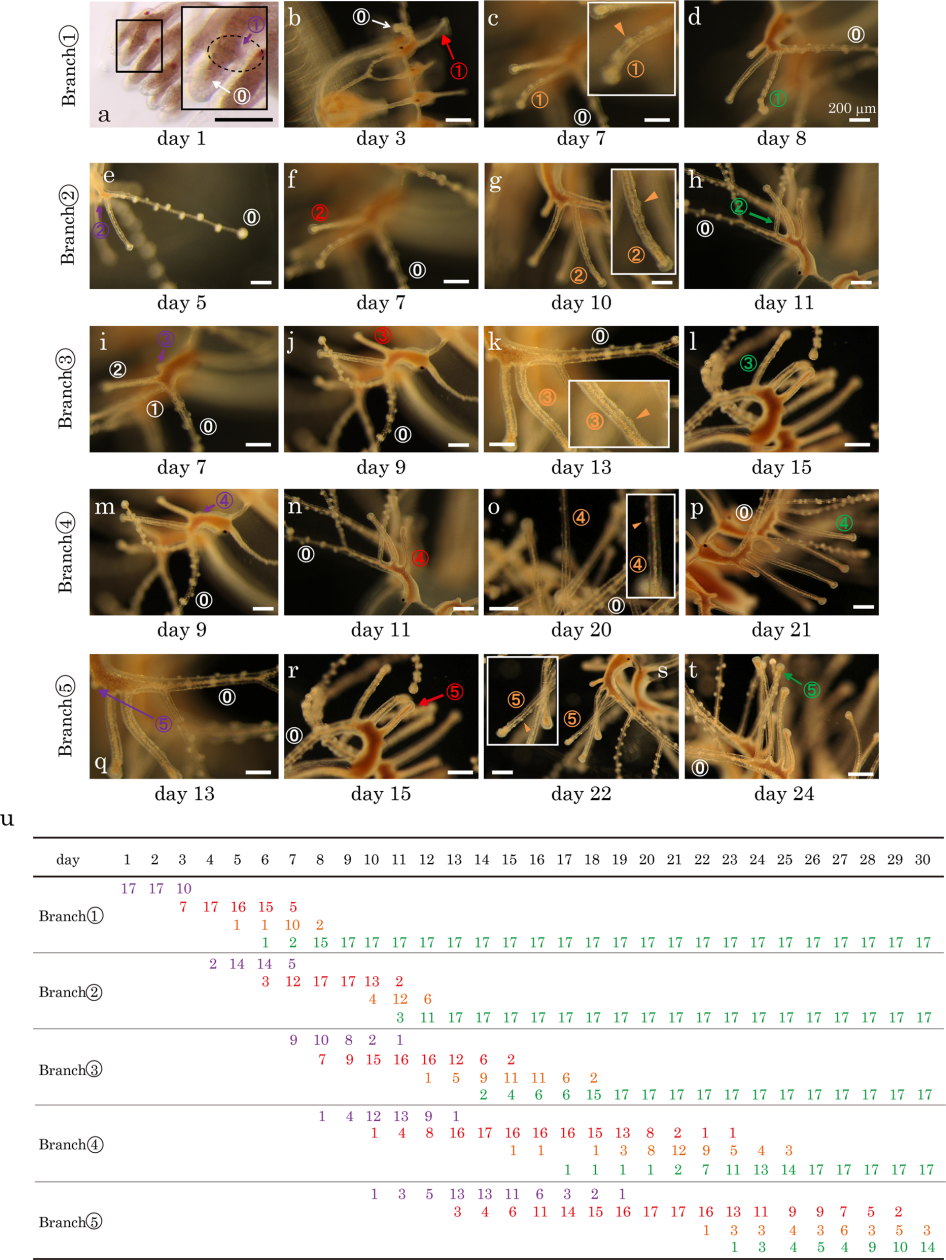
Functional changes in the tentacle branches. **a**–**t** A series of images following the same tentacle branches (Branch① in **a**–**d**, Branch② in **e**–**h**, Branch③ in **i**–**l**, Branch④ in **m**–**p**, and Branch⑤ in **q**–**t**) demonstrating that they change their functions from landing to hunting. The branches were numbered in the same way as in Fig. 2. Whether a branch sticks to the tip of a tungsten needle was used as the criterion for possessing functional adhesive organs; whether it captures Artemia Nauplius was used to evaluate whether it has functional nematocysts. Branches without either function are indicated by purple, those with functional adhesive organs only by red, those with functional adhesive organs and functional nematocysts by orange, and those with only functional nematocysts by green. The insets are blown-up images of the branches shown in orange, in which the positions of the nematocyst clusters are indicated by arrowheads. The same individual was used for the images in Figs. 2 and 6; therefore, there are several pairs of identical pictures (2a and 6a, 2c and 6b, 2e and 6e, 2g and 6i, and 2h and 6d). Scale bars: 200 μm. (u) A table showing when Branches ① to ⑤ acquired their functions in 17 cases/tentacles. Each number represents how many numbered branches had which of the four functional states, purple, red, orange or green, on a given day

To understand how the two types of tentacle branches, including the adhesive organs (landing branch) and the nematocyst clusters (hunting branch), are created during tentacle growth, we continually monitored the tentacle growth every 24 h and recorded branch identities for 30 days. We categorized branch identities into four groups depending on whether the branch had neither, both, or one of the functional adhesive organs and functional nematocysts. We observed that new branches formed without either feature (indicated by purple in Fig. 6) but soon developed functional adhesive organs at their tips (red in Fig. 6). This was consistently true for the branches observed during the 30-day period (from Branch① to Branch⑤); we did not observe any new branches that became hunting branches first. The newly formed landing branches then acquired functional nematocysts at various positions along the length of the branches without any particular pattern (orange in Fig. 6). They gradually lost their adhesive organs and became hunting branches possessing only functional nematocyst clusters (green in Fig. 6). These observations suggest that there is a functional transition from landing to hunting abilities within the same branches as they develop.

**Fig. 7:**
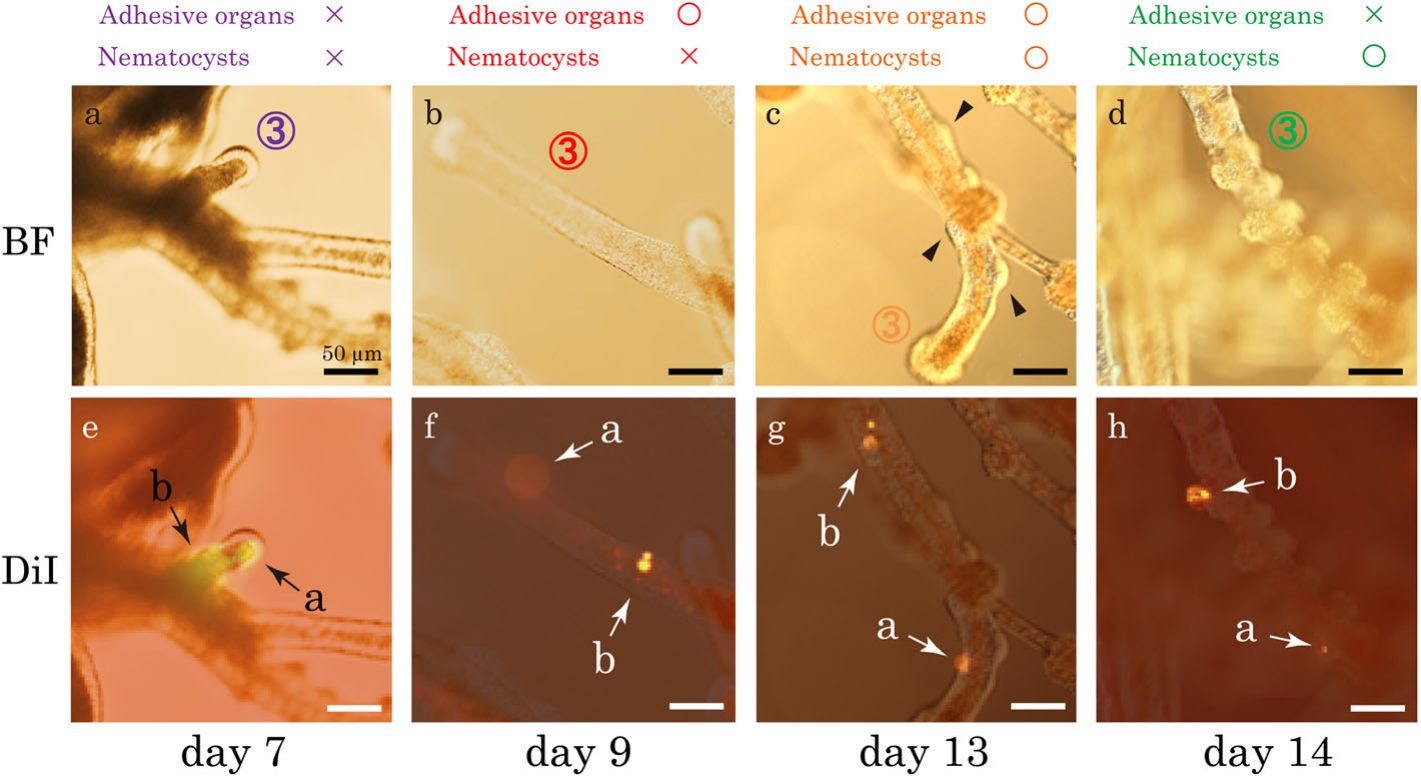
Following the same branch with DiI labeling. Two spots (a and b) on the third branch (Branch③) were labeled with DiI on Day 7. Images of the labeled branch on different days (Day 7 in **a** and **e**, Day 9 in **b** and **f**, Day 13 in **c** and **g** and Day 14 in **d** and **h**) with bright field (**a**-**d**) and with DiI fluorescence microscopy (**e**-**h**). Note that function of branch changed as development proceeds. The functional states of the branch are indicated above the images in colors. Three independent labeling experiments were performed, and the same results were obtained. Scale bars: 50 μm

To further support this notion, we tracked individual branches with DiI labelling and monitored their branch identity changes during tentacle growth. We labelled two spots on the third branches (Branch③) with DiI on Day 7 and continuously observed them until Day 14. On day 7, the third branches did not have either function (Figs. 6u, 7a). We confirmed that the same branches indeed changed their function from landing to hunting during their development (Fig. 7).

**Fig. 8:**
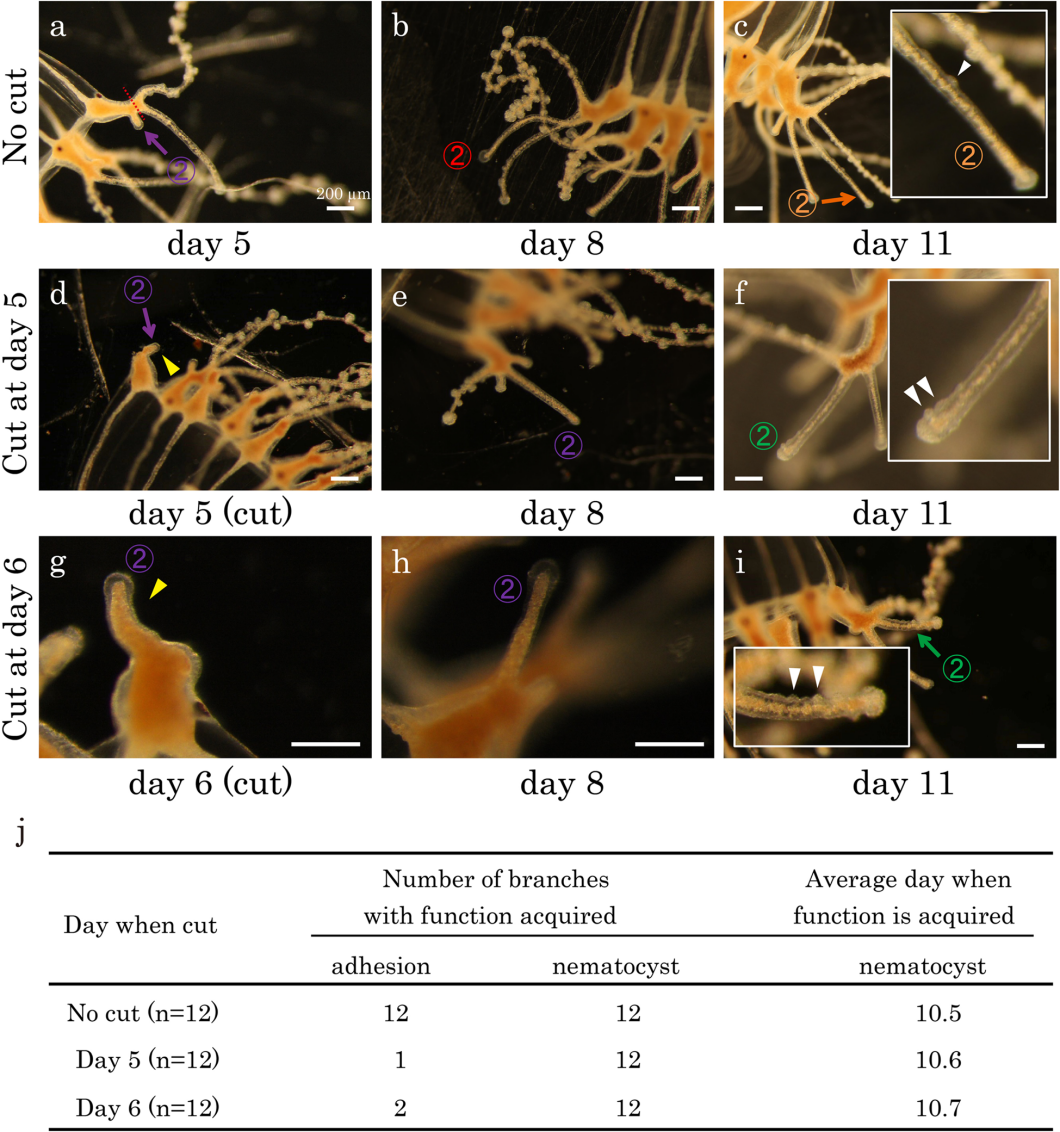
The formation of nematocysts without adhesive organ formation. **a**–**i** Images of the second branch (Branch②) on different days without (**a**-**c**) and with (**d**-**i**) ablations. For ablation, a distal part of the medusa tentacle including the first branch (Branch①) was cut off on Day 5 (**d**) or Day 6 (**g**) as indicated by the red broken line in **a**. The second branch (Branch②) remained with the main body, as shown by the yellow arrowheads in **d** and **g**. The functional status of branches are indicated by colored numbers in the same way as Fig. 6. The insets are blown-up images of Branch② with nematocyst clusters denoted with white arrowheads. By Day 8, the “no cut” Branch② acquired functional adhesive organs (**b** and Fig. 6u), but the ablated second branches did not (**e** and **h**). However, they formed functional nematocysts on the same day as “no cut” Branch② (**c**, **f** and **i**). Scale bars: 200 μm. **j** A table showing the number of branches with either function that were formed by Day 11, and on the day on which functional nematocysts were acquired for the first time

To understand how branch differentiation is regulated, we carried out an ablation experiment. In this experiment, we cut off the distal branch with a razor (broken red line in Fig. 8a) on either Day 5 or 6 to eliminate effects of the distal part of the tentacles, including the first branches, on the second branches. We then examined whether the remaining second branches differentiated into landing and/or hunting branches. On Day 5 and 6 when we performed surgery, the second branches did not have either function (Figs. 6u, 8d, g). We found that most of the second branches failed to form functional adhesive organs on Day 8 when they were cut on Day 5 (92% (*n* = 12) or on Day 6 (83% (*n* = 12))(Fig. 8e, h, j), while they normally do form adhesive organs (Figs. 6u, 8b). Interestingly, the same branches formed functional nematocysts on Day 11 according to the normal schedule (Fig. 6u) without experiencing adhesive organ formation (100% (*n* = 12) for branches cut on Day 5 and Day 6) (Fig. 8f, i, j). These results suggest that formation of functional adhesive organs requires the distal part of the tentacles and is dispensable for functional nematocyst differentiation.

## Discussion

### Medusa tentacle branching

Our observations of medusa tentacle branch formation in the jellyfish *C. pacificum* reveal features of branch patterning and morphogenesis that are common to other well-studied branching systems in animal species, as well as ones that are unique to *C. pacificum*. While *C. pacificum* branched tentacles appear to be an elaborate structure, we found that they form through repeated applications of a simple rule: branching at the proximal part of the main tentacle. This mechanism of repeating a simple rule is widely used in other branching systems [7, 17, 19, 20, 23], and thus may represent a fundamental mechanism for generating complex structures, such as branched organs, across a wide range of animal species including non-bilaterians. However, we also found that the branching of *C. pacificum* tentacles differed from that in other branching systems, including those found in corals and colonial hydroids [16–18], in that it occurs at the proximal end of the branching structure. The proximal region of medusa tentacles has been shown to be the site of active cell proliferation in *Aurelia* and *Clytia* jellyfish species [30, 31], which suggests that cell proliferation may be involved in the proximal branching in *C. pacificum*. In examples of branching from mammalian and *Drosophila* models of airway formation and angiogenesis, branches form at the tips of branching tissues [21, 22]. This may be because the tissues are growing branches to find all possible target cells, thus branching at sites of cell searches, possibly in response to signals from these cells, is likely more efficient. Medusa tentacles, on the other hand, can flexibly move their branches by muscle contraction, even after the branch architecture has been established. Further, the adhesive branches may contribute to the unique method of tentacle branching in *C. pacificum*. The adhesive branches, which extend off of the adaxial side of the proximal main tentacles (Fig. 1b), enable the medusa to “stand up” on a substratum, such as seagrass. This allows for the medusa to secure a space between the mouth and the substratum, while the distally located nematocyst branches deliver prey into the mouth. However, as the main tentacles extend in length, the adhesive branches shift too far away to contribute to standing and no longer serve their original function. It might therefore be more efficient to recycle these established branches into hunting branches than to form new branches at distal regions. Despite the different branching methods between *C. pacificum* and other animals, the resulting branch structures are advantageous for expanding the epithelial surface areas and maximizing functions.

### Acquisition of adhesive organs and nematocysts

The results from our ablation experiments indicate that the acquisition of nematocysts does not depend on formation of adhesive organs (Fig. 8). We speculate that this is because the nutrition-finding function of nematocysts was prioritized over adhesive organ formation after surgical removal of the distal parts of tentacles containing nematocyst clusters (Fig. 8a). During tentacle growth, nematocyst clusters appear on the main tentacle as early as on Day 2, even before the adhesive organ is formed for the first time on the first branch (Fig. 6u). In support of this notion, we found that limiting the amount of *Artemia* Nauplius prey to two individuals per day, or every other day, enhanced the formation of functional nematocysts in the absence of adhesive organ formation (0% with an excess amount of the prey every day (*n* = 12); 25% with two prey per day (n = 12); 61.1% with two prey every other day (n = 12)). Interestingly, however, functional nematocysts did not form earlier in the ablated tentacles than in controls (Fig. 8); thus, the timing of nematocyst acquisition may be tightly regulated.

### RTK signaling and mesoderm origin

Branch formation occurs through local cellular movements, such as cell migration, proliferation, rearrangement, and deformation, which generate new branch buds [20–23]. We found that tentacle branching in *C. pacificum* may be initiated by extension of the epidermal epithelial cells along the apico-basal axis. This observation highlights a possibly important and conserved role of regulating epithelial cell shape in branch formation among a wide range of animals covering both non-bilaterian and bilaterian animals. In the mammalian pancreas and salivary gland, branch bud cells have a characteristic columnar shape [32, 33]. In stolons of hydroids, a plate of columnar ectodermal cells is formed at the site of branching [17].

At the molecular level, many of the cellular behaviors involved in branch formation require receptor tyrosine kinase (RTK) signaling [20–22]. For example, FGF signaling is required for specification of leading cells in cell migration in the *Drosophila* trachea [34] and mammary gland [35] and for regionalized cell proliferation in the mouse salivary gland [10], vascular endothelial growth factor (VEGF) signaling is required for leading cell specification in mammalian retinal blood vessels [36], and glial cell-derived neurotrophic factor (GDNF) signaling is required for cell proliferation in the mouse kidney [37]. The ligands for these RTKs are produced in the mesenchyme, which surrounds the core structure of branched organs made of epithelial cells. In this study, we found that inhibition of MEK in *C. pacificum* led to the absence of branch formation in the tentacles, suggesting that medusa tentacle branch formation in this species also requires RTK signaling. However, we note that jellyfish are diploblastic animals without mesoderm. Although this is debatable, as there is bilaterian-like striated muscle in the sub-umbrella region of most hydrozoan medusae and the striated muscle originates in the entocodon cell mass which develops between the ectoderm and endoderm [38, 39], to our knowledge there are no mesoderm-like cells in the tentacle region. Therefore, it is of particular interest to determine the source ligand for RTK signaling in tentacle branch formation.

In relation to the absence of mesoderm in the medusa tentacles, we would also like to note that the tentacle branches extend out towards the apical side of the epithelial layers. This contrasts with branching morphogenesis in *Drosophila* and mammals, where branches grow into the mesenchyme located on the basal side of the epithelial layers [20–23]. In this sense, the medusa tentacles as well as stolons [17] in hydrozoa species, may be more comparable to plant roots in terms of cellular processes of branch formation. Plant roots also extend their branches out towards the external environment and regionalized cell proliferation is involved in their branch formation [40].

### Branched tentacles as a new trait in evolution

*Cladonema pacificum* belongs to the family Cladonematidae, which is characterized by a number of synapomorphic features including branched medusa tentacles with adhesive organs [27]. Therefore, studying *Cladonema* tentacle branch formation could provide clues to understand how a new trait might have been acquired in the course of evolution. Another genus that belongs to the family Cladonematidae, *Staurocladia*, also has branched tentacles [27, 41], which, unlike *C. pacificum*, branch only once. It would be interesting to examine how the *Staurocladia* species prevent further branch formation. The regulation of RTK signaling might be involved in this inter-genus difference.

The *Staurocladia* medusa main tentacles have nematocyst clusters with single branches bearing adhesive organs extending off the adaxial side of the main tentacle. Unlike *C. pacificum,* the branches do not seem to change their functions. In our study of *C. pacificum* medusae at Day 7, at which time the third branches (Branch③) are first observed (Fig. 2j) and the second branches (Branch②) have only adhesive organs (Fig. 6u), we tried to eliminate the effect of the third branches by cutting them on Day 7. Although this cutting resulted in regeneration of branches at the cut site on the next day, we cut them again, mimicking the situation in *Staurocladia*, which lacks any younger branches. We then examined whether the second branches change their function to nematocyst branches after the third branch ablation. We found that their function shifted following the normal time course (100%, *n* = 7), suggesting that the presence or absence of third branches does not determine whether the second branches change their function. Therefore, the lack of functional changes in *Staurocladia* branches may not be due to the absence of younger branches. Continued study of these two closely related species would further explain the developmental and evolutionary aspects of tentacle branch formation that may possibly apply to other species without branched tentacles.

## Conclusions

In the present study, we described details of branching patterns in the medusa tentacles in a jellyfish species. Despite the phylogenetic distance between cnidaria and more complex and well-studied animals such as *Drosophila* and mammals, we found that the cnidarian species use branching mechanisms in similar ways including the repeated use of a simple rule and the involvement of RTK signaling. On the other hand, we also found unique mechanisms specific to the jellyfish. Accordingly, the current study provides us a unique opportunity to further study the fundamental mechanisms of branching morphogenesis across a wide range of animal species and to discover novel principles of creating branched structures.
